# Social determinants of health and premature death among adults in the USA from 1999 to 2018: a national cohort study

**DOI:** 10.1016/S2468-2667(23)00081-6

**Published:** 2023-06

**Authors:** Joshua D Bundy, Katherine T Mills, Hua He, Thomas A LaVeist, Keith C Ferdinand, Jing Chen, Jiang He

**Affiliations:** Department of Epidemiology, Tulane University School of Public Health and Tropical Medicine, New Orleans, LA, USA; Tulane University Translational Science Institute, New Orleans, LA, USA; Department of Epidemiology, Tulane University School of Public Health and Tropical Medicine, New Orleans, LA, USA; Tulane University Translational Science Institute, New Orleans, LA, USA; Department of Epidemiology, Tulane University School of Public Health and Tropical Medicine, New Orleans, LA, USA; Tulane University Translational Science Institute, New Orleans, LA, USA; Department of Health Policy and Management, Tulane University School of Public Health and Tropical Medicine, New Orleans, LA, USA; Department of Medicine, Tulane University School of Medicine, New Orleans, LA, USA; Department of Epidemiology, Tulane University School of Public Health and Tropical Medicine, New Orleans, LA, USA; Tulane University Translational Science Institute, New Orleans, LA, USA; Department of Medicine, Tulane University School of Medicine, New Orleans, LA, USA; Department of Epidemiology, Tulane University School of Public Health and Tropical Medicine, New Orleans, LA, USA; Tulane University Translational Science Institute, New Orleans, LA, USA; Department of Medicine, Tulane University School of Medicine, New Orleans, LA, USA

## Abstract

**Background:**

Racial and ethnic disparities in mortality persist in the US population. We studied the contribution of social determinants of health (SDoH) to racial and ethnic disparities in premature death.

**Methods:**

A nationally representative sample of individuals aged 20–74 years who participated in the US National Health and Nutrition Examination Survey (NHANES) between 1999 and 2018 were included. Self-reported SDoH (employment, family income, food security, education, access to health care, health insurance, housing instability, and being married or living with a partner) were collected in each survey cycle. Participants were categorised into four groups of race and ethnicity: Black, Hispanic, White, and other. Deaths were ascertained from linkage to the National Death Index with follow-up until 2019. Multiple mediation analysis was used to assess simultaneous contributions of each individual SDoH to racial disparities in premature all-cause mortality.

**Findings:**

We included 48 170 NHANES participants in our analyses, consisting of 10 543 (21·9%) Black participants, 13 211 (27·4%) Hispanic participants, 19 629 (40·7%) White participants, and 4787 (9·9%) participants of other racial and ethnic groups. Mean survey-weighted age was 44·3 years (95% CI 44·0–44·6), 51·3% (50·9–51·8) of participants were women, and 48·7% (48·2–49·1) were men. 3194 deaths before age 75 years were recorded (930 Black participants, 662 Hispanic participants, 1453 White participants, and 149 other participants). Black adults had significantly higher premature mortality than other racial and ethnic groups (p<0·0001): premature death rates per 100 000 person-years were 852 (95% CI 727–1000) for Black adults, 445 (349–574) for Hispanic adults, 546 (474–630) for White adults, and 521 (336–821) for other adults. Unemployment, lower family income, food insecurity, less than high school education, no private health insurance, and not being married nor living with a partner were significantly and independently associated with premature death. Dose–response associations were observed between cumulative number of unfavourable SDoH and premature all-cause mortality: hazard ratios (HRs) were 1·93 (95% CI 1·61–2·31) for those with one unfavourable SDoH, 2·24 (1·87–2·68) for those with two, 3·98 (3·34–4·73) for those with three, 4·78 (3·98–5·74) for those with four, 6·08 (5·06–7·31) for those with five, and 7·82 (6·60–9·26) for those with six or more unfavourable SDoH (p<0·0001 for linear trend). After adjusting for SDoH, HRs for premature all-cause mortality for Black adults compared with White adults decreased from 1·59 (1·44–1·76) to 1·00 (0·91–1·10), suggesting complete mediation of this racial difference in mortality.

**Interpretation:**

Unfavourable SDoH are associated with increased rates of premature death and contribute to differences between Black and White racial groups in premature all-cause mortality in the US population. Innovative public health policies and interventions targeting SDoH are needed to reduce premature deaths and health disparities in this population.

**Funding:**

US National Institutes of Health.

## Introduction

Racial, ethnic, and socioeconomic disparities in all-cause mortality and life expectancy persist in the US population.^1–4^ Increases in life expectancy plateaued in 2010, and, although racial and ethnic gaps in life expectancy have narrowed, data from 2019 suggest stagnating gains.^4,5^ In 2019, Black people had a more than 20% greater rate of all-cause mortality than White people.^6^ However, race is a social, rather than biological, construct, and is not causally related to mortality but reflects underlying social factors.^7^ On average, compared with White adults, Black adults have higher levels of poverty and unemployment.^8^ Therefore, the importance of social determinants of health (SDoH) in addressing health inequities is increasingly recognised.^9^ The SDoH framework reflects a comprehensive assessment of the health effects of the “conditions in the environments where people are born, live, learn, work, play, worship, and age” and shapes public health initiatives such as the US Healthy People 2030 initiative, which seeks to achieve health equity and improve the health and wellbeing of all people.^10,11^

Although the associations of individual SDoH with various adverse health outcomes have been reported,^12–20^ most previous studies have focused on a single social determinant or socioeconomic status. In 2017, a meta-analysis of more than 1·7 million individuals reported that low socioeconomic status was associated with 42% greater all-cause mortality compared with high socioeconomic status.^18^ Educational attainment, poverty, and lack of health insurance might explain, in part, increased mortality across some sociodemographic groups of adults in the USA.^19,20^ However, previous reports have not comprehensively and simultaneously studied the effects of multiple domains of SDoH on mortality and their contributions to racial and ethnic differences in premature death in the US population.

The objectives of this study were to examine the prospective associations of multiple SDoH with premature all-cause mortality, and to investigate SDoH as mediators of racial and ethnic disparities in premature all-cause mortality in US adults.

## Methods

### Study participants

The National Health and Nutrition Examination Survey (NHANES) uses stratified, multistage probability sampling methods to select a series of nationally representative samples of non-institutionalised US adults in 2-year cycles since 1999–2000.^21^ We included ten survey cycles from 1999–2000 to 2017–18 and excluded individuals missing unique identifiers sufficient for linkage to the National Death Index. In the primary analyses, we evaluated premature death, defined as death occurring before 75 years of age, which was the approximate mean age of death in the USA throughout the study period.^6^ In a secondary analysis, we included participants aged 20 years or older, with no upper age limit. NHANES protocols were approved by the ethics review board of the National Center for Health Statistics and all participants provided written informed consent for survey participation and for their data to be used for health-related statistical research and linked to vital statistics (eg, the National Death Index).

### Data collection and SDoH

In each 2-year survey, standardised questionnaires were used to collect information on age, gender, race and ethnicity, and SDoH. Because Asian was not listed as a separate race and ethnicity category in NHANES until 2011–12, we grouped participants into four race and ethnicity categories: non-Hispanic Black, Hispanic, non-Hispanic White, and other.

We reviewed standardised NHANES questionnaires to identify SDoH variables across the five Healthy People 2030 domains: economic stability, education access and quality, health-care access and quality, neighbourhood and built environment, and social and community context.^10^ We included eight SDoH available in each NHANES cycle from 1999 to 2018 (the 2017–18 cycle was the latest cycle linked to mortality follow-up) in the main analyses: employment status, family income-to-poverty ratio calculated by dividing family income by a family size-specific poverty threshold, food security (based on responses to ten questions about aspects of food unavailability, with full availability defined as no affirmative responses), education level, health-care access (whether the participant had a routine facility where they accessed health care or health advice, other than an emergency room), health insurance status (private *vs* government or no insurance), home ownership, and being married or living with a partner. Detailed descriptions of SDoH-related questions are available in the appendix (p 5). Although social support and crowded housing were also identified as SDoH variables, they were not considered further because social support was unavailable in survey cycles from 2009 to 2018, and living in crowded housing was uncommon among NHANES participants (6·1%) and showed a weak and imprecise association with premature all-cause mortality (hazard ratio [HR] 1·23 [95% CI 0·99–1·53]).

We investigated the associations of several single SDoH measures with premature all-cause mortality using various categorisations and with adjustment for age, gender, and race and ethnicity (appendix p 6). For multiple mediation analysis and evaluation of a cumulative burden of SDoH, SDoH were dichotomised on the basis of conventional cutoff points (appendix p 6).^10,11,22–24^ Because the continuous family income-to-poverty ratio showed a linear association with premature death, it was dichotomised at the median (300%). We defined an unfavourable SDoH as the level within an SDoH category that was associated with a higher premature mortality rate.

We explored the effect of an accumulation of unfavourable SDoH within individuals on premature all-cause mortality by summing the eight dichotomised SDoH, assigning a value of 0 for each favourable and 1 for each unfavourable level, to create a cumulative SDoH variable. Because only a small proportion of participants reported six, seven, or eight unfavourable SDoH, they were combined, yielding a cumulative SDoH variable ranging from zero to six or more.

### Ascertainment of deaths

Deaths were identified by linkage of NHANES participants to the National Death Index, with follow-up until Dec 31, 2019.^25^ In the primary analyses, we evaluated premature death, defined as death occurring before 75 years of age, which was the approximate mean age of death of US adults throughout the study period.^6^ We also investigated the associations of SDoH with cardiovascular, cancer-related, and other causes of death. In a secondary analysis, we evaluated all deaths regardless of age of occurrence. In additional sensitivity analyses, we evaluated associations of SDoH with premature death before 65, 70, and 80 years of age.

### Statistical analysis

All statistical analyses accounted for the complex survey design and yielded population-weighted estimates representative of the non-institutionalised US population from 1999 to 2018. We estimated proportions of participants in each SDoH category overall and by race and ethnicity (Black, Hispanic, White, and other). We assessed the pairwise correlations among the dichotomous SDoH variables using Cramér’s V.^26^ Kaplan-Meier analysis was used to plot cumulative mortality overall and in gender subgroups and race and ethnicity subgroups using age as the timescale.^27^ We standardised absolute mortality rates to the 2010 US Census population distribution of age and gender, and expressed them as deaths per 100 000 person-years.

We estimated the associations of SDoH with all-cause mortality using Cox proportional hazards regression, with age as the timescale.^27,28^ We accounted for left truncation given that participants were included in NHANES only if they survived long enough to have a baseline examination. Additionally, models were stratified by birth cohort using 10-year intervals to control for effects of survey year, cohort, and calendar period. We estimated associations using two models: (1) adjusted for gender and race and ethnicity; and (2) adjusted for gender and race and ethnicity and additionally including the other SDoH to identify independent, direct associations. Additionally, we used Poisson regression to estimate incidence rate differences adjusted for age, gender, and race and ethnicity. We plotted HRs of the cumulative SDoH variable to visualise whether its relationship with premature death was linear or non-linear. Additionally, we evaluated the consistency of associations between SDoH and premature death in subgroups of gender and race and ethnicity. No significant interactions were detected; therefore, the primary analyses are presented in participants overall. However, given that men had significantly higher premature mortality than women, Kaplan-Meier plots are presented overall and stratified by gender.

We investigated the extent to which differences in premature all-cause mortality between Black and White populations and between Hispanic and White populations are explained by SDoH using multiple mediation analysis with the R package mma, using methods and applications published previously.^29,30^ Because all-cause mortality was not significantly different between Hispanic, White, and other populations, we focused on differences between Black and White populations. The multiple mediation analysis included all eight SDoH factors as mediators simultaneously to assess their contributions to racial and ethnic differences in premature mortality, assuming that these factors are potential intermediates on the causal pathway from race and ethnicity to mortality, and could be intervened upon to mitigate racial and ethnic differences in mortality. We estimated the relative contributions (ie, proportions mediated) of each SDoH factor in explaining the racial and ethnic difference in all-cause mortality. Further details of the multiple mediation analysis are available in the appendix (p 2).

Given that we included participants with baseline SDoH assessments over 20 years, there is potential for secular trends in SDoH prevalence and associations with all-cause mortality. Therefore, we did a sensitivity analysis by two 10-year survey periods (1999–2008 and 2009–18) to check the consistency of SDoH distributions and associations with premature all-cause mortality over time.

We used SAS software (version 9.4) and R software (version 4.2.1) with survey analysis procedures to account for the complex sampling design in all analyses, yielding estimates nationally representative of the US population. We used fully efficient fractional imputation of missing questionnaire data, in which each individual’s missing data were imputed from multiple donor individuals with non-missing data.^31^ Less than 3% of values were missing for all variables except family income-to-poverty ratio (9·6% missing). Point estimates were calculated using imputation-adjusted survey weights appropriate for combining survey cycles, while standard errors and 95% CIs were generated using jackknife replicate weights. All statistical tests were two-sided, and p<0·05 was considered statistically significant.

### Role of the funding source

The funder of the study had no role in study design, data collection, data analysis, data interpretation, or writing of the report.

## Results

After excluding 142 individuals who did not have unique identifiers to allow linkage to the National Death Index, our analyses included 48 170 NHANES participants aged 20–74 years, consisting of 10 543 (21·9%) Black participants, 13 211 (27·4%) Hispanic participants, 19 629 (40·7%) White participants, and 4787 (9·9%) participants of other racial and ethnic groups. The mean survey-weighted age was 44·3 years (95% CI 44·0–44·6), 51·3% (50·9–51·8) participants were women, and 48·7% (48·2–49·1) were men (table 1). Most SDoH were moderately correlated with each other (appendix p 7). Compared with White participants, Black and Hispanic participants had lower levels of employment, family income, food security, educational attainment, health-care access, private health insurance, and home ownership. A lower proportion of Black participants were married or living with a partner compared with people from all other racial and ethnic groups.

**Figure 1:**
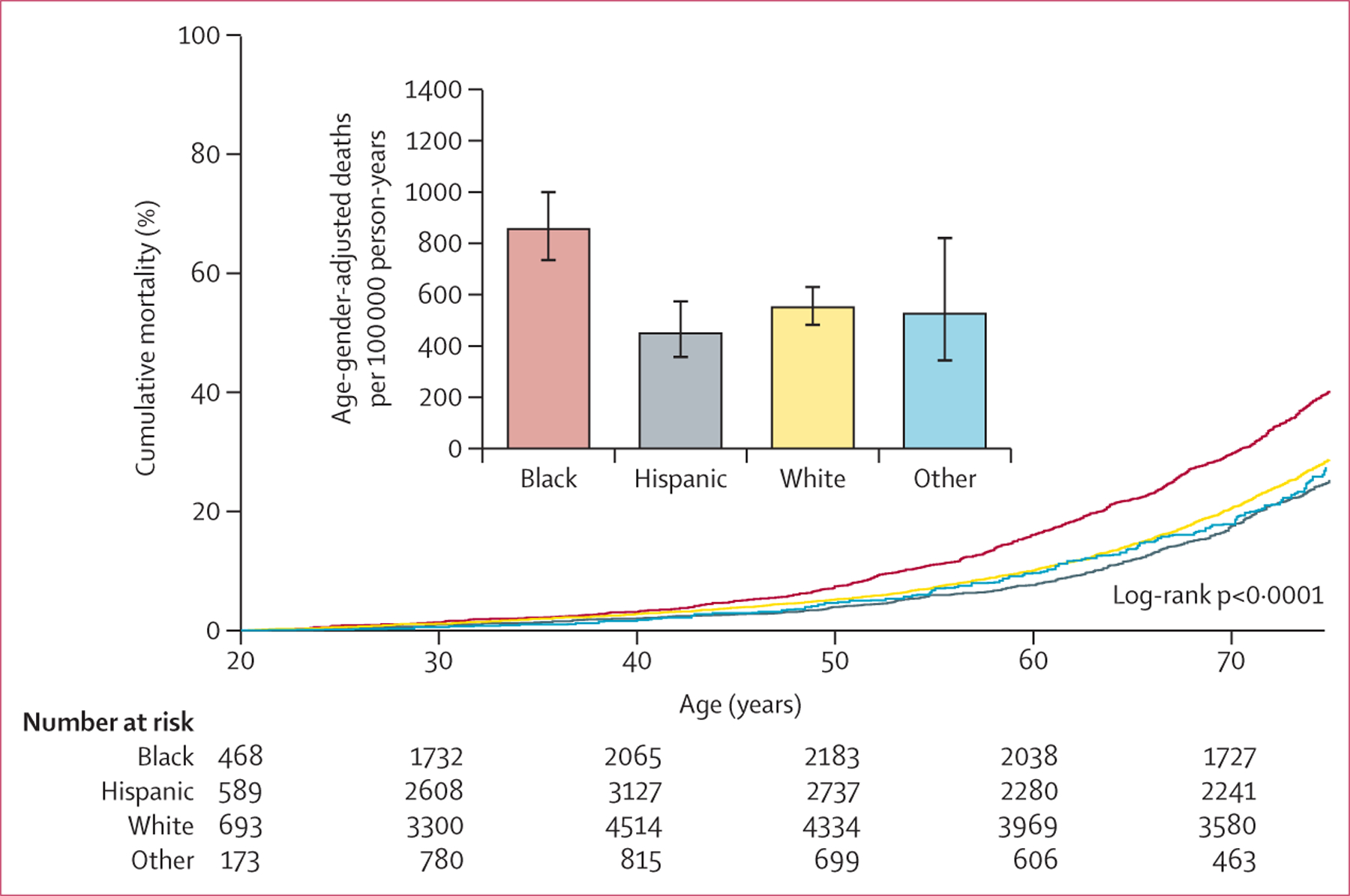
Premature all-cause mortality in US adults aged 20–74 years by gender and race and ethnicity Kaplan-Meier curves show cumulative mortality by age and race and ethnicity. Numbers at risk are unweighted observed frequencies. Cumulative mortality rates were estimated with use of imputation-adjusted survey weights. Bar chart shows age-gender-adjusted deaths per 100 000 person-years (standardised to the 2010 US Census population) by race and ethnicity; error bars are 95% CIs.

3194 deaths before age 75 years were recorded (930 Black participants, 662 Hispanic participants, 1453 White participants, and 149 other participants) during a median 9·7 years (IQR 5·3–14·5) of follow-up. Black adults had a higher premature death rate compared with other racial and ethnic groups in men and women (figure 1; appendix p 3). The premature mortality rate was similar across racial and ethnic groups until approximately 40 years of age, when it began to diverge, characterised by higher rates in Black participants compared with those in other racial and ethnic groups. After standardising absolute mortality rates to the distribution of age and gender in the 2010 US Census population, premature death rates per 100 000 person-years were 852 (95% CI 727–1000) for Black adults, 445 (349–574) for Hispanic adults, 546 (474–630) for White adults, and 521 (336–821) for other adults (figure 1). Patterns were similar in men and women, although men had a higher premature mortality rate (appendix p 3).

In models stratified by birth cohort and adjusted for gender and race and ethnicity, an unfavourable level of each SDoH was significantly associated with higher premature mortality (table 2). After additional adjustment for other SDoH, unemployment, family income-to-poverty ratio less than 300%, food insecurity, less than high school education, government health insurance or no health insurance, and not being married nor living with a partner remained significantly associated with increased premature mortality. Multivariable-adjusted HRs for SDoH significantly associated with premature mortality ranged from 1·20 (95% CI 1·09–1·33) for food insecurity to 2·11 (1·91–2·33) for unemployment. Directions and magnitudes of associations of SDoH with premature mortality were mostly similar by race and ethnicity (appendix p 8). Associations were similar in direction and magnitude for cardiovascular, cancer-related, or other specific causes of death, although slightly weaker for non-cardiovascular and non-cancer-related deaths (appendix p 9).

**Figure 2:**
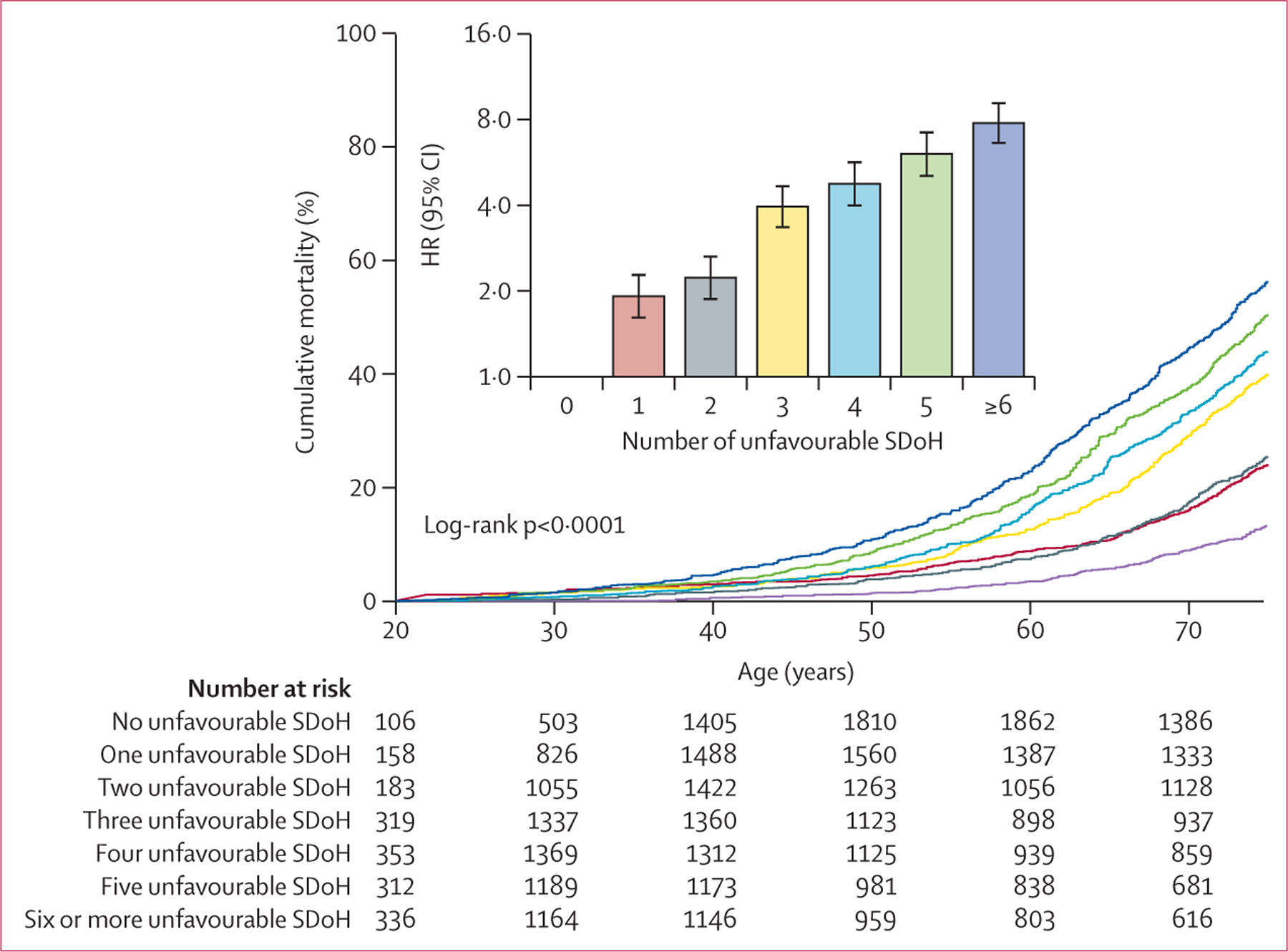
Premature all-cause mortality and HRs in US adults aged 20–74 years according to the number of unfavourable SDoH Kaplan-Meier curves show cumulative mortality by age and number of unfavourable SDoH. Numbers at risk are unweighted observed frequencies. Cumulative mortality rates were estimated with use of imputation-adjusted survey weights. Bar chart shows HRs of premature all-cause mortality associated with number of unfavourable SDoH, adjusted for age, gender, and race and ethnicity; error bars are 95% CIs. HR=hazard ratio. SDoH=social determinants of health.

Linear dose–response associations were observed between cumulative SDoH and premature mortality (figure 2). Overall, compared with those with no unfavourable SDoH, HRs were 1·93 (95% CI 1·61–2·31) for those with one unfavourable SDoH, 2·24 (1·87–2·68) for those with two, 3·98 (3·34–4·73) for those with three, 4·78 (3·98–5·74) for those with four, 6·08 (5·06–7·31) for those with five, and 7·82 (6·60–9·26) for those with six or more unfavourable SDoH (p<0·0001 for linear trend). Patterns were similar in men and women (appendix p 4).

The age-gender-adjusted HR for premature mortality comparing Black with White participants was 1·59 (95% CI 1·44–1·76; table 3). After further adjusting for all SDoH, the HR was diminished and became statistically non-significant (1·00 [0·91–1·10]). Family income-to-poverty ratio less than 300% (23·6%), government health insurance or no health insurance (18·8%), not being married nor living with a partner (18·1%), unemployment (15·9%), less than high school education (10·6%), and food insecurity (9·4%) showed the highest relative contributions to the difference between Black and White participants in premature mortality. The age-gender-adjusted HR for premature mortality comparing Hispanic with White participants was 0·84 (0·72–0·98; appendix p 10). After further adjusting for all SDoH, the HR decreased to 0·50 (0·42–0·60).

In a secondary analysis, we included all NHANES participants aged 20 years or older (n=54 927) and evaluated associations of SDoH with all-cause mortality at any age. 9175 deaths were documented (1798 Black participants, 1556 Hispanic participants, 5493 White participants, and 328 other participants). Distributions of SDoH among racial and ethnic groups were similar to those in the main analysis (appendix pp 11–12). In addition, associations of SDoH with all-cause mortality were similar to, although slightly weaker than, those in the main analysis (appendix p 13).

In sensitivity analyses using varying cutoff points for premature death (65, 70, and 80 years of age), associations of SDoH with all-cause mortality were consistent (appendix p 14). In a sensitivity analysis using two 10-year survey periods, the distribution of SDoH among racial and ethnic groups (appendix pp 15–16) and associations of SDoH with premature all-cause mortality (appendix p 17) were mostly similar over time.

## Discussion

This study shows that Black and Hispanic adults in the USA had higher prevalence of unfavourable SDoH compared with White adults. In addition, unfavourable SDoH were associated with higher premature all-cause mortality. The number of unfavourable SDoH showed a dose–response association with premature mortality, with having six or more unfavourable SDoH increasing mortality by a factor of 7·8 compared with having no unfavourable SDoH. Furthermore, after adjusting for SDoH, there was no longer a difference between Black and White adults in premature all-cause mortality. These findings suggest that racial differences in premature all-cause mortality are completely explained by differences in SDoH.

The persistence of racial and ethnic disparities in premature death are well documented.^1–4^ Beydoun and colleagues^19^ and Luo and colleagues^20^ previously showed that racial and ethnic disparities in mortality among participants in NHANES III (1988–94) might be partly, but not completely, explained by SDoH including income-to-poverty ratio, education, and health insurance status. For example, Beydoun and colleagues found that the top contributors to differences between Black and White populations were family poverty-to-income ratio (40% mediated) and education (19% mediated),^19^ which were consistent with the results of Luo and colleagues (62% mediated by income).^20^ Our work corroborates these results for income and education and expands upon them by considering the simultaneous contributions^30^ of eight factors across all five SDoH domains of Healthy People 2030 and using US nationally representative NHANES data from 1999 to 2018, with follow-up until 2019. In addition to our finding that the prevalence of unfavourable SDoH explained disparities in premature mortality between Black and White adults, we found that accounting for SDoH increased the differences in mortality between Hispanic and White individuals, with premature mortality being significantly lower in Hispanic than White people, consistent with the so-called Hispanic paradox reported by Beydoun and colleagues^19^ and others, which might be due to unmeasured protective social behaviours and cultural values.^32^ These findings implicate SDoH as major drivers of premature death and racial health disparities.

Compared with White adults, Black adults were more likely to have unfavourable levels of all SDoH. Black adults were 1·6 times more likely than White adults to have a family income-to-poverty ratio below 300%, which was associated with almost 50% greater premature mortality. Furthermore, unemployment was associated with more than two-times greater premature mortality. Together, the traditional socioeconomic factors of education, family income, and unemployment explained around 50% of difference between Black and White adults in premature mortality. An important corollary is that nearly 50% of the difference in premature mortality was explained by other factors, most prominently health insurance status, being married or living with a partner, and food security. Although these additional factors might be affected by the availability of economic resources, our findings suggest independent associations beyond education and income. Policy changes targeting socioeconomic factors represent a promising pathway to improving health equity,^33^ but simultaneous consideration of all five Healthy People 2030 SDoH domains^10^ is warranted. Being on a government health insurance programme or having no insurance showed a large relative contribution to disparities between Black and White adults in premature mortality. Insurance status might be highly correlated with traditional socioeconomic measures such as employment, but our findings suggest an independent association. It is unlikely that having government health insurance directly increases mortality risk. Indeed, Medicaid expansion is associated with reductions in all-cause mortality and associated disparities.^34^ However, policy makers should consider the circumstances that cause individuals to be on government insurance programmes and why those with private insurance retain a substantial survival advantage, and should identify pathways to equitably insure all those living in the USA.

We also found that being married or living with a partner (18·1% relative contribution) and food insecurity (9·4% relative contribution) explained large portions of disparities between Black and White people in premature mortality. In our study sample, Black adults were around 35% less likely to be married or living with a partner compared with White adults. Being married or living with a partner is associated with improved survival, possibly due to reduced health-threatening behaviours or greater social support.^35^ Black adults were also around twice as likely as White adults to have food insecurity, which was independently associated with premature all-cause mortality among US adults in our study and others.^24,36^ Although the exact mechanisms by which food insecurity increases the risk of all-cause mortality are unknown, proposed explanations include insufficient intake of essential nutrients or increased reliance on unhealthy food choices.^36,37^ Additional research is needed to understand these mechanisms and to develop appropriate public health programmes and interventions, in addition to and beyond food assistance programmes.^37^ We found that having six or more unfavourable SDoH was associated with approximately eight times the risk for premature mortality among US adults. Conversely, those with no unfavourable SDoH had low mortality rates that persisted until 70 years of age, highlighting the importance of seeking an optimal level of SDoH for all people. The magnitudes of association with all-cause mortality we observed for cumulative unfavourable SDoH greatly exceed those of similar variables constructed from lifestyle and biological factors.^38^ Although unfavourable SDoH tend to cluster in individuals,^24^ our results suggest not only independent associations of several single SDoH factors, but a cumulative effect of multiple unfavourable SDoH on premature mortality. The previously developed social deprivation index, which relies on SDoH ascertained at the area or neighbourhood level, has been associated with adverse health outcomes including obesity and diabetes.^39,40^ Our approach ascertained similar information to social deprivation indices, but based on individuals’ responses to questionnaires. Although our approach weighted the eight SDoH equally to evaluate a simple linear dose–response relationship, future research could explore the development of a polysocial risk score weighting each SDoH on the basis of its relative strength of association, pending availability of suitable validation datasets.^41,42^

Our study has several limitations. First, some important SDoH were not widely available in NHANES, including family wealth, experienced racism, discrimination, violence, and social support. These and other factors, including early-life and family SDoH, might be contributing causes of racial disparities in premature death. Additionally, because we had only one baseline assessment of SDoH, we could not evaluate the effect of changing SDoH over time, nor time-varying confounding. However, the eight SDoH measured at baseline only were able to completely explain the disparity between Black and White adults in premature death. Second, there is potential for secular trends, given that SDoH were collected over a span of 20 years of baseline evaluations. However, we found that their prevalence and associations with premature death were similar across 10-year survey periods. Third, although premature death is one of the most important population-level health indicators, data on disease burden were unavailable. Fourth, we cannot rule out the possibility of residual confounding. Additionally, misclassification in self-reported SDoH categories is possible, although likely to be non-differential with respect to mortality and, thus, resulting in attenuation of results toward the null. Fifth, NHANES enrolled non-institutionalised individuals. Because Black adults are disproportionately incarcerated in the USA due to structural racism,^8^ our findings of differences between Black and White adults might be underestimated. Sixth, the National Death Index only captures deaths that occurred in the USA or its territories; thus, participants who died outside the USA would be misclassified as alive. Finally, more information on additional categories of race and ethnicity was not available due to sample size and NHANES questionnaire design. Therefore, the other category for race and ethnicity might include a heterogeneous mix of individuals at lower risk and higher risk (eg, Indigenous Americans).^4^

In conclusion, unfavourable SDoH are more common among Black than White adults, are strongly associated with premature death, and completely explain differences between Black and White adults in premature death. Our findings, which can be extrapolated to the general population of the USA, provide support for an increasingly recognised public health framework.^10,11^ Although this observational study cannot definitively establish causality, our findings suggest that holistically targeting unfavourable SDoH and their underlying causes could reverse negative trends in life expectancy in US adults and close associated racial gaps in mortality.^43,44^ Addressing SDoH will require concerted efforts to initiate structural, multilevel policy interventions targeting the underlying causes of death among all people, and particularly those from historically marginalised populations.^13,33,43^ Long-standing racial oppression and intergenerational, area-level propagation of structural racism are such underlying causes; thus, place-based, multisector, equity-oriented initiatives with government support are necessary to target SDoH improvement.^8^ For example, Racial Equity Here is composed of hundreds of diverse community organisations working together to target racial and health equity. Finally, future research should examine the effects of SDoH on global disparities in mortality and identify major determinants for intervention.

## Supplementary Material

1

## Figures and Tables

**Table 1:** Characteristics of study participants aged 20–74 years by race and ethnicity

	All participants (n=48 170)	Self-reported race and ethnicity			
		Black (n=10 543)	Hispanic (n=13 211)	White (n=19 629)	Other* (n=4787)
Age, years	44·3 (44·0–44·6)	42·6 (42·2–43·0)	39·8 (39·4–40·3)	45·8 (45·4–46·2)	42·7 (42·0–43·4)
Gender					
Female	24 994 (51·3% [50·9–51·8])	5464 (54·9% [54·1–55·7])	7001 (49·8% [48·9–50·6])	10 046 (50·9% [50·3–51·5])	2483 (52·3% [50·8–53·9])
Male	23 176 (48·7% [48·2–49·1])	5079 (45·1% [44·3–45·9])	6210 (50·2% [49·4–51·1])	9583 (49·1% [48·5–49·7])	2304 (47·7% [46·1–49·2])
Employment status					
Employed, student, or retired	36 189 (79·8% [79·1–80·5])	7725 (73·7% [72·4–75·0])	9455 (75·1% [74·1–76·2])	15 323 (82·2% [81·3–83·1])	3686 (77·5% [75·9–79·1])
Unemployed	11 941 (20·2% [19·5–20·9])	2810 (26·3% [25·0–27·6])	3747 (24·9% [23·8–25·9])	4294 (17·8% [16·9–18·7])	1090 (22·5% [20·9–24·1])
Family income-to-poverty ratio					
≥300%	16 684 (50·2% [48·7–51·7])	3079 (32·8% [30·8–34·7])	2624 (24·7% [23·1–26·4])	8998 (59·0% [57·1–60·9])	1983 (48·7% [45·6–51·8])
<300%	27 096 (49·8% [48·3–51·3])	6422 (67·2% [65·3–69·2])	8950 (75·3% [73·6–76·9])	9436 (41·0% [39·1–42·9])	2288 (51·3% [48·2–54·4])
Food security†					
Full security	32 937 (78·0% [77·1–78·9])	6757 (65·1% [63·4–66·8])	7335 (57·5% [55·6–59·4])	15 295 (84·6% [83·7–85·6])	3550 (78·4% [76·1–80·7])
Marginal, low, or very low security	13 921 (22·0% [21·1–22·9])	3505 (34·9% [33·2–36·6])	5439 (42·5% [40·6–44·4])	3914 (15·4% [14·4–16·3])	1063 (21·6% [19·3–23·9])
Education level					
High school graduate or higher	35 502 (83·3% [82·5–84·2])	7935 (77·3% [75·9–78·8])	6659 (58·4% [56·8–59·9])	16 858 (89·6% [88·6–90·6])	4050 (85·5% [84·0–87·0])
Less than high school	12 605 (16·7% [15·8–17·5])	2594 (22·7% [21·2–24·1])	6531 (41·6% [40·1–43·2])	2751 (10·4% [9·4–11·4])	729 (14·5% [13·0–16·0])
Regular health-care access					
At least one regular health-care facility	36 868 (78·8% [78·0–79·6])	8138 (75·6% [74·3–76·9])	9048 (64·8% [63·3–66·4])	16 118 (82·9% [82·0–83·8])	3564 (74·2% [72·2–76·2])
None or emergency room	11 298 (21·2% [20·4–22·0])	2405 (24·4% [23·1–25·7])	4163 (35·2% [33·6–36·7])	3510 (17·1% [16·2–18·0])	1220 (25·8% [23·8–27·8])
Type of health insurance					
Private	25 522 (63·9% [62·8–65·0])	5137 (50·3% [48·7–52·0])	5063 (41·0% [39·5–42·6])	12 581 (71·7% [70·4–73·0])	2741 (60·0% [57·5–62·5])
Government or none	22 077 (36·1% [35·0–37·2])	5241 (49·7% [48·0–51·3])	7973 (59·0% [57·4–60·5])	6883 (28·3% [27·0–29·6])	1980 (40·0% [37·5–42·5])
Home ownership					
Own home	28 458 (66·7% [65·4–68·1])	5104 (47·1% [45·1–49·1])	6875 (49·9% [47·4–52·5])	13 787 (74·6% [73·3–75·8])	2692 (59·7% [56·4–63·1])
Rent home or other arrangement	18 803 (33·3% [31·9–34·6])	5243 (52·9% [50·9–54·9])	6011 (50·1% [47·5–52·6])	5613 (25·4% [24·2–26·7])	1936 (40·3% [36·9–43·6])
Marital status					
Married or living with a partner	29 249 (64·4% [63·4–65·3])	4675 (44·2% [42·7–45·7])	8618 (64·4% [63·0–65·7])	12 711 (67·6% [66·6–68·6])	3245 (66·5% [64·5–68·5])
Not married nor living with a partner	18 414 (35·6% [34·7–36·6])	5758 (55·8% [54·3–57·3])	4430 (35·6% [34·3–37·0])	6708 (32·4% [31·4–33·4])	1518 (33·5% [31·5–35·5])
Cumulative SDoH variable‡					
0	6449 (21·8% [20·8–22·9])	874 (9·1% [8·1–10·1])	786 (7·1% [6·4–7·8])	4096 (27·8% [26·5–29·0])	693 (17·0% [15·0–18·9])
1	6770 (20·1% [19·4–20·8])	1167 (12·7% [11·9–13·6])	1098 (10·1% [9·2–10·9])	3729 (23·7% [22·8–24·7])	776 (18·8% [16·8–20·7])
2	6325 (15·8% [15·2–16·3])	1323 (14·2% [13·4–15·0])	1364 (12·5% [11·7–13·3])	2915 (16·5% [15·8–17·3])	723 (17·8% [16·1–19·5])
3	6212 (13·3% [12·8–13·8])	1426 (15·4% [14·6–16·3])	1721 (15·1% [14·3–15·9])	2402 (12·3% [11·6–12·9])	663 (15·7% [14·3–17·2])
4	6262 (12·0% [11·5–12·5])	1575 (17·6% [16·7–18·5])	1997 (17·8% [16·9–18·7])	2058 (9·5% [9·0–10·1])	632 (14·2% [12·6–15·8])
5	5409 (9·2% [8·8–9·7])	1379 (15·4% [14·5–16·4])	2051 (18·3% [17·3–19·2])	1554 (6·1% [5·6–6·6])	425 (9·8% [8·7–11·0])
6 or more	5139 (7·8% [7·3–8·2])	1466 (15·6% [14·4–16·7])	2191 (19·2% [18·0–20·4])	1208 (4·0% [3·6–4·5])	274 (6·7% [5·6–7·8])

Data are mean (95% CI) or n (% [95% CI]); absolute numbers are unweighted, observed values; means, percentages, and 95% CIs are population-weighted and derived from imputed data. SDoH=social determinants of health.

* Includes non-Hispanic Asian, American Indian or Alaska Native, and Native Hawaiian or Pacific Islander.

† Full food security indicates no affirmative responses to the individual Food Security Survey Module questions, and marginal or lower food security indicates one or more affirmative responses.

‡ Constructed by assigning a value of 0 for each favourable and 1 for each unfavourable level, and summing the eight dichotomised SDoH; a higher number indicates the presence of more unfavourable SDoH.

**Table 2:** Associations of SDoH with premature death in US adults aged 20–74 years

	Deaths/total participants	Total person-years of follow-up	Incidence rate (95% CI), per 1000 person-years	Adjusted* incidence rate difference (95% CI), per 1000 person-years	HR (95% CI), stratified by birth cohort	
					Adjusted for gender and race and ethnicity	Adjusted for gender, race and ethnicity, and other SDoH
**Employment status**						
Employed, student, or retired	1814/36 189	366 477	4·23 (4·00–4·46)	0 (ref)	1 (ref)	1 (ref)
Unemployed	1380/11 941	114 592	10·90 (10·05–11·74)	6·84 (6·01–7·68)	3·06 (2·79–3·36)	2·11 (1·91–2·33)
**Family income-to-poverty ratio**						
≥300%	730/16 684	175 775	3·95 (3·63–4·27)	0 (ref)	1 (ref)	1 (ref)
<300%	2163/27 096	264 219	7·19 (6·78–7·59)	3·50 (3·00–3·99)	2·45 (2·21–2·73)	1·48 (1·31–1·67)
**Food security**						
Full security	2097/32 937	343 990	5·02 (4·74–5·29)	0 (ref)	1 (ref)	1 (ref)
Marginal, low, or very low security	1003/13 921	123 891	7·68 (7·09–8·27)	4·32 (3·61–5·03)	2·18 (1·98–2·40)	1·20 (1·09–1·33)
**Education level**						
High school graduate or higher	1941/35 502	348 714	4·74 (4·44–5·05)	0 (ref)	1 (ref)	1 (ref)
Less than high school	1241/12 605	132 102	9·22 (8·48–9·97)	4·07 (3·16–4·98)	2·05 (1·81–2·32)	1·33 (1·18–1·51)
**Regular health-care access**						
At least one regular health-care facility	2569/36 868	372 109	5·64 (5·35–5·93)	0 (ref)	1 (ref)	1 (ref)
None or emergency room	625/11 298	109 295	5·07 (4·46–5·68)	1·33 (0·51–2·16)	1·29 (1·13–1·48)	0·98 (0·86–1·11)
**Type of health insurance**						
Private	1302/25 522	267 823	4·13 (3·85–4·40)	0 (ref)	1 (ref)	1 (ref)
Government or none	1844/22 077	207 170	8·28 (7·71–8·86)	4·02 (3·42–4·63)	2·38 (2·15–2·64)	1·35 (1·20–1·53)
**Home ownership**						
Own home	1903/28 458	291 470	5·53 (5·21–5·86)	0 (ref)	1 (ref)	1 (ref)
Rent home or other arrangement	1239/18 803	183 073	5·51 (5·11–5·91)	2·45 (1·88–3·02)	1·71 (1·55–1·90)	1·05 (0·95–1·15)
**Marital status**						
Married or living with a partner	1617/29 249	295 931	4·76 (4·46–5·06)	0 (ref)	1 (ref)	1 (ref)
Not married nor living with a partner	1509/18 414	176 712	6·95 (6·49–7·42)	2·90 (2·44–3·37)	1·90 (1·73–2·08)	1·44 (1·32–1·58)

Number of deaths, total participants, and total person-years of follow-up in each category are unweighted observed frequencies. Incidence rates (95% CIs), incidence rate differences (95% CIs), and HRs (95% CIs) were estimated with imputation-adjusted survey weights and jackknife replicate weights. HR=hazard ratio. SDoH=social determinants of health.

* Adjusted for age, gender, and race and ethnicity.

**Table 3:** Mediation by SDoH of the difference between Black and White racial groups in premature death among US adults aged 20–74 years

	HR (95% CI)	Relative contribution, % (95% CI)*
Stratified by birth cohort and adjusted for gender		
Black *vs* White race	1·59 (1·44 to 1·76)	··
Stratified by birth cohort and adjusted for gender and other SDoH		
Black *vs* White race	1·00 (0·91 to 1·10)	··
Unemployed *vs* employed, student, or retired	2·15 (1·91 to 2·43)	15·9% (10·0 to 21·9)
Family income-to-poverty ratio <300% *vs* ≥300%	1·45 (1·27 to 1·66)	23·6% (13·6 to 33·7)
Marginal, low, or very low food security *vs* full food security	1·21 (1·07 to 1·37)	9·4% (3·2 to 15·6)
Less than high school education *vs* high school graduate or higher	1·43 (1·25 to 1·65)	10·6% (5·2 to 16·0)
No regular health-care access *vs* at least one regular health-care facility	1·00 (0·86 to 1·15)	0·0% (–2·5 to 2·5)
No private health insurance *vs* private insurance	1·45 (1·26 to 1·67)	18·8% (9·5 to 28·2)
No home ownership *vs* home ownership	1·04 (0·94 to 1·15)	2·4% (–4·4 to 9·2)
Not married nor living with a partner *vs* married or living with a partner	1·37 (1·23 to 1·53)	18·1% (10·8 to 25·4)

HR=hazard ratio. SDoH=social determinants of health.

* Percentage of the racial difference in all-cause premature mortality explained by each factor in the multivariable mediation model.

## Research in context

### Evidence before this study

We searched PubMed from database inception to April 7, 2023, using the Medical Subject Headings terms “social determinants of health” OR “socioeconomic factors” AND “health status disparities” OR “healthcare disparities” OR “race factors” AND “mortality, premature” OR “mortality” OR “death” OR “life expectancy” AND “prospective studies” OR “cohort studies” OR “longitudinal studies” AND “United States” AND “adult” OR “aged” OR “young adult”, with no date or language restrictions. The search retrieved 110 relevant publications. We also reviewed the cited references of retrieved publications. Previous work shows that racial and ethnic disparities in life expectancy have persisted in the USA for decades. Black adults have significantly higher mortality than White adults. In addition, compared with White adults, Black adults have higher levels of unfavourable social determinants of health (SDoH), including poverty, unemployment, and low educational attainment. Previous studies conducted among US adults have shown that unfavourable SDoH are strongly associated with various health outcomes and might explain, in part, racial differences in mortality. However, most previous studies have focused on socioeconomic status and contributions of single factors to racial differences in mortality. There is increasing recognition that SDoH across all domains (economic stability, education access and quality, health-care access and quality, neighbourhood and built environment, and social and community context) are important for population health and affect health disparities, but this association has not been comprehensively evaluated in the US general population.

### Added value of this study

We quantified the associations of unfavourable SDoH with premature death among 48 170 individuals aged 20–74 years from the US general population, and evaluated the extent to which multiple SDoH explain racial differences in premature death. To date, this is the most comprehensive analysis of SDoH in a nationally representative sample of the US general population. We used multiple mediation analysis to show that the eight SDoH in our study completely explained differences between Black and White adults in premature death. Hazard ratios for premature all-cause mortality for Black adults compared with White adults decreased from 1·59 (95% CI 1·44–1·76) to 1·00 (0·91–1·10) after adjusting for SDoH, which completely mediated racial differences in mortality. In addition, unemployment, lower family income, food insecurity, less than high school education, no private health insurance, and not being married nor living with a partner were significantly and independently associated with premature death. Moreover, we observed a dose–response relationship between the cumulative number of unfavourable SDoH and premature death, with six or more unfavourable SDoH increasing mortality by a factor of 7·8.

### Implications of all the available evidence

Our results suggest that unfavourable SDoH are associated with higher premature death and explain racial disparities in all-cause mortality in the US population. Taken together with previous findings, SDoH are major risk factors for premature death and underlying causes of racial health disparities. Future research should examine the effect of SDoH on global disparities in mortality and identify major determinants for intervention. Addressing the SDoH identified by our study will require concerted efforts to initiate structural, multilevel policy interventions targeting the underlying causes of death among all people, and particularly those from historically marginalised populations.
