# Supplementary material for: Social determinants of health and premature death among adults in the USA from 1999 to 2018: a national cohort study
Source: Lancet Public Health. Author manuscript; Available in PMC 2023 Jul 15. (PMC10349537; doi:10.1016/S2468-2667(23)00081-6)
Supplement: 1 [file NIHMS1904461-supplement-1.pdf]

THE LANCET
Public Health

Supplementary appendix

This appendix formed part of the original submission and has been peer reviewed. We post it as supplied by the authors.

Supplement to: Bundy JD, Mills KT, He H, et al. Social determinants of health and premature death among adults in the USA from 1999 to 2018: a national cohort study. *Lancet Public Health* 2023; **8:** e422–31.

Supplementary Appendix

Social Determinants of Health and Premature Death Among US Adults

Appendix Methods

Figure S1. Premature All-cause Mortality in US Adults Aged 20 to 74 Years by Gender, and Race and Ethnicity.

Figure S2. Premature All-cause Mortality and Hazard Ratios in US Adults Aged 20 to 74 Years According to the Number of Unfavorable Social Determinants of Health.

Table S1: US Centers for Disease Control Healthy People 2030 Social Determinants of Health in NHANES 1999-2018.

Table S2: Candidate Social Determinants of Health and Associations with Premature All-cause Mortality in US Adults Aged 20 to 74 Years, NHANES 1999-2018.

Table S3. Pairwise Correlations between Social Determinants of Health in US Adults Aged 20 to 74 Years, NHANES 1999-2018.

Table S4. Associations of Social Determinants of Health with Premature All-cause Mortality in US Adults Aged 20 to 74 Years by Race/ethnicity, NHANES 1999-2018.

Table S5. Associations of Social Determinants of Health with Premature All-cause Mortality in US Adults Aged 20 to 74 Years by Cause of Death, NHANES 1999-2018.

Table S6. Mediation of the Hispanic-White Difference in Premature Death by Social Determinants of Health in US Adults Aged 20 to 74 Years, National Health and Nutrition Examination Surveys 1999-2018.

Table S7. Characteristics of Study Participants Aged 20 Years or Older by Race/ethnicity, NHANES 1999-2018.

Table S8. Associations of Social Determinants of Health with All-cause Mortality in US Adults Aged 20 Years or Older, NHANES 1999-2018.

Table S9: Associations of Social Determinants of Health with Premature All-cause Mortality in US Adults by Varying Age Cut-off Points, NHANES 1999-2018.

Table S10: Characteristics of Study Participants Aged 20 to 74 Years by 10-year Survey Cycle Groups, NHANES 1999-2018.

Table S11: Associations of Social Determinants of Health with Premature All-cause Mortality in US Adults Aged 20 to 74 Years by 10-year Survey Cycle Groups, NHANES 1999-2018.

Appendix Methods

Figure. Path diagram to investigate mediation effects of SDOH.

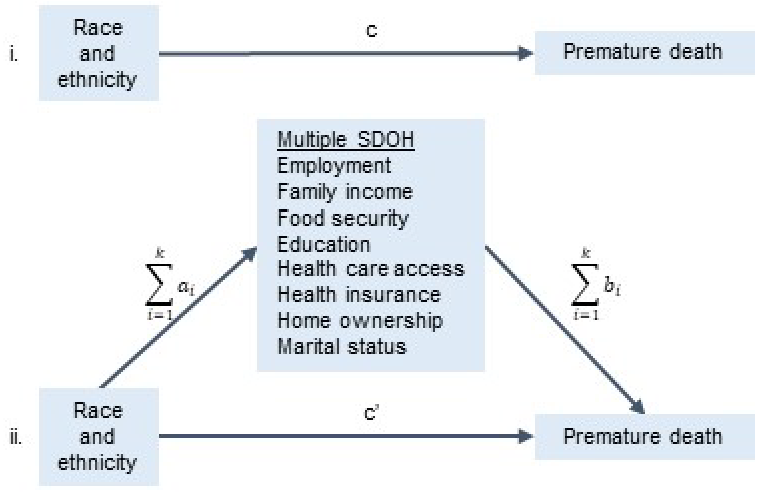

We investigated the extent to which Black-White and Hispanic-White differences in premature all-cause mortality are explained by SDOH using multiple mediation analysis via the R package *mma* with methods and applications published previously.[1,2] Specifically, we considered the counterfactual scenario of the distribution of mediators among the disadvantaged group (e.g., Black adults) being equal to the advantaged group (e.g., White adults). The multiple mediation analysis included all eight SDOH factors as mediators simultaneously to assess their contributions to racial/ethnic differences in premature mortality, assuming they are potential intermediates on the causal pathway from race to mortality. We estimated the relative contributions (i.e., proportions mediated) of each SDOH factor in explaining the racial/ethnic difference in all-cause mortality.

The multiple mediation method is a non/semiparametric approach that allows the comparison of relative effects carried by different mediators on the outcome of interest. The model includes a main exposure (i.e., race or ethnicity), multiple mediators (i.e., all SDOH of interest), and an outcome (i.e., time to premature death). The model progresses through 2 algorithms to derive the estimates shown in the path diagram Figure, namely the direct effect of race/ethnicity on premature death (c'), the individual indirect effects of exposure on outcome mediated ($a_i + b_i$), and the total effect of race/ethnicity on premature death.[1,2] In the first algorithm, the total effect is estimated by generating the joint effects of the mediators at each level of race/ethnicity, and then summed. In the second algorithm, the direct effect of race/ethnicity not mediated is estimated using the samples generated by algorithm 1 and random permutation. The combined approach is based on a random resampling approach, and repeated 100 times to generate average results for the whole multiple mediation model.

The multiple mediation model relies on three basic assumptions: 1) no unmeasured confounding for the exposure-outcome relationship; 2) no unmeasured confounding for the mediator-outcome relationship; and 3) mediator M_i is not causally prior to other mediators M_{-i}. While the possibility for residual confounding is unavoidable in observational research (e.g., due to family history SDOH), we have included a comprehensive set of SDOH across all domains. Additionally, it is possible some mediators (e.g., education) are causally prior to others (e.g., family income-poverty ratio). However, we jointly model the SDOH together so although individual direct effects of mediators must be interpreted with caution, we can appropriately model the direct effect of race/ethnicity on premature death that does not proceed via the SDOH.[3]

We calculated the relative effects (i.e., proportion mediated) for each single SDOH by taking the indirect effect for each respective SDOH and dividing by the total effect. The R package *mma* accommodates weighting, and jackknife replicate weights were used to account for the complex survey design.[4]

Appendix References

1 Yu Q, Li B. mma: An R Package for Mediation Analysis with Multiple Mediators. *J Open Res Softw* 2017; **5**: 11.

2 Yu Q, Wu X, Li B, Scribner RA. Multiple mediation analysis with survival outcomes: With an application to explore racial disparity in breast cancer survival. *Stat Med* 2019; **38**: 398–412.

3 Yu Q, Fan Y, We X. General Multiple Mediation Analysis With an Application to Explore Racial Disparities in Breast Cancer Survival. *J Biom Biostat* 2014; **5**: 1–9.

4 Canty AJ, Davison AC. Resampling-Based Variance Estimation for Labour Force Surveys. *J R Stat Soc Ser D (The Stat* 1999; **48**: 379–91.

Figure S1. Premature All-cause Mortality in US Adults Aged 20 to 74 Years by Gender, and Race and Ethnicity.

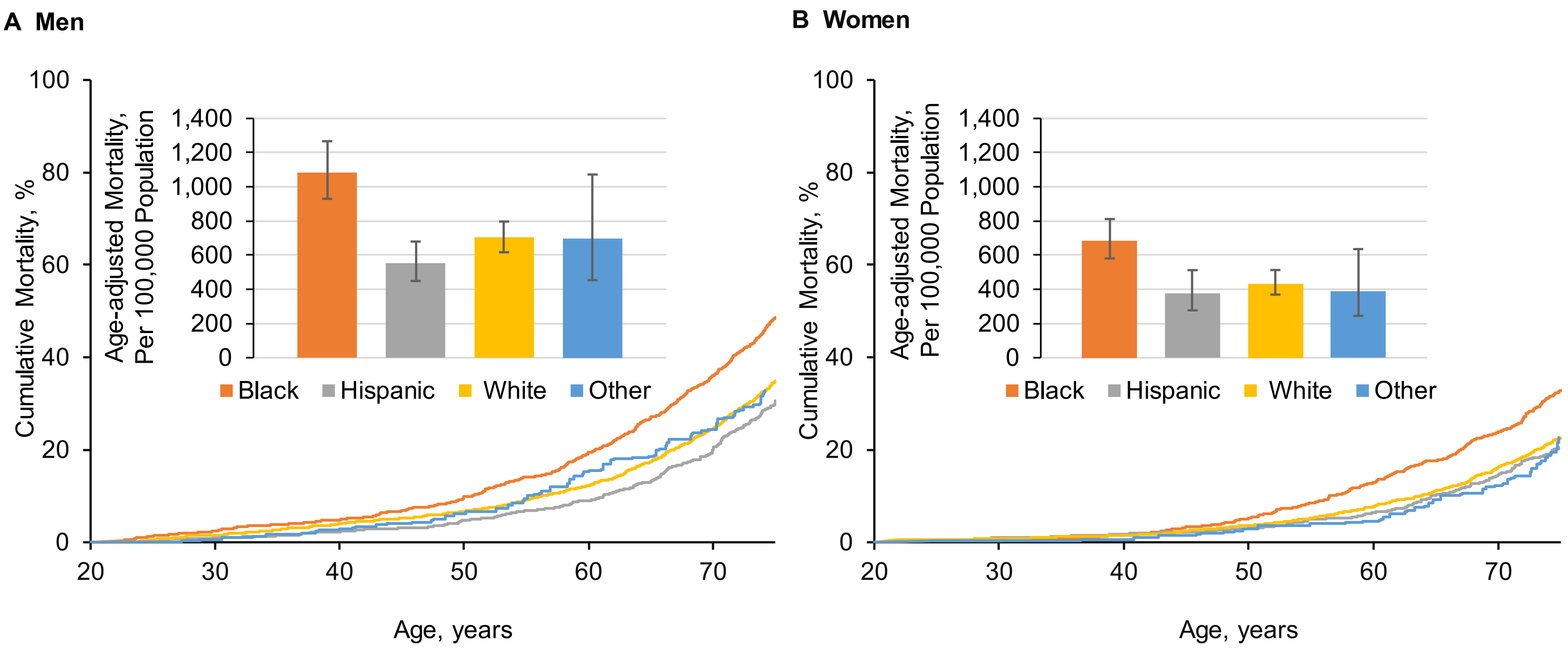

Kaplan-Meier curves show cumulative mortality rates by age, and race and ethnicity. Bar charts show age-adjusted mortality rates per 100,000 population (standardized to the 2010 US Census population) and 95% confidence intervals by race and ethnicity. (A) Among men (n=23,176), the annual age-adjusted premature mortality rates (95% CIs) per 100,000 population for Black, Hispanic, White, and other adults were 1085 (930-1267), 552 (449-680), 701 (617-797), and 696 (454-1072), respectively. (B) Among women (n=24,994), the annual age-adjusted premature mortality rates (95% CIs) per 100,000 population for Black, Hispanic, White, and other adults were 686 (581-812), 373 (277-512), 435 (369-513), 389 (245-636), respectively.

Figure S2. Premature All-cause Mortality and Hazard Ratios in US Adults Aged 20 to 74 Years According to the Number of Unfavorable Social Determinants of Health.

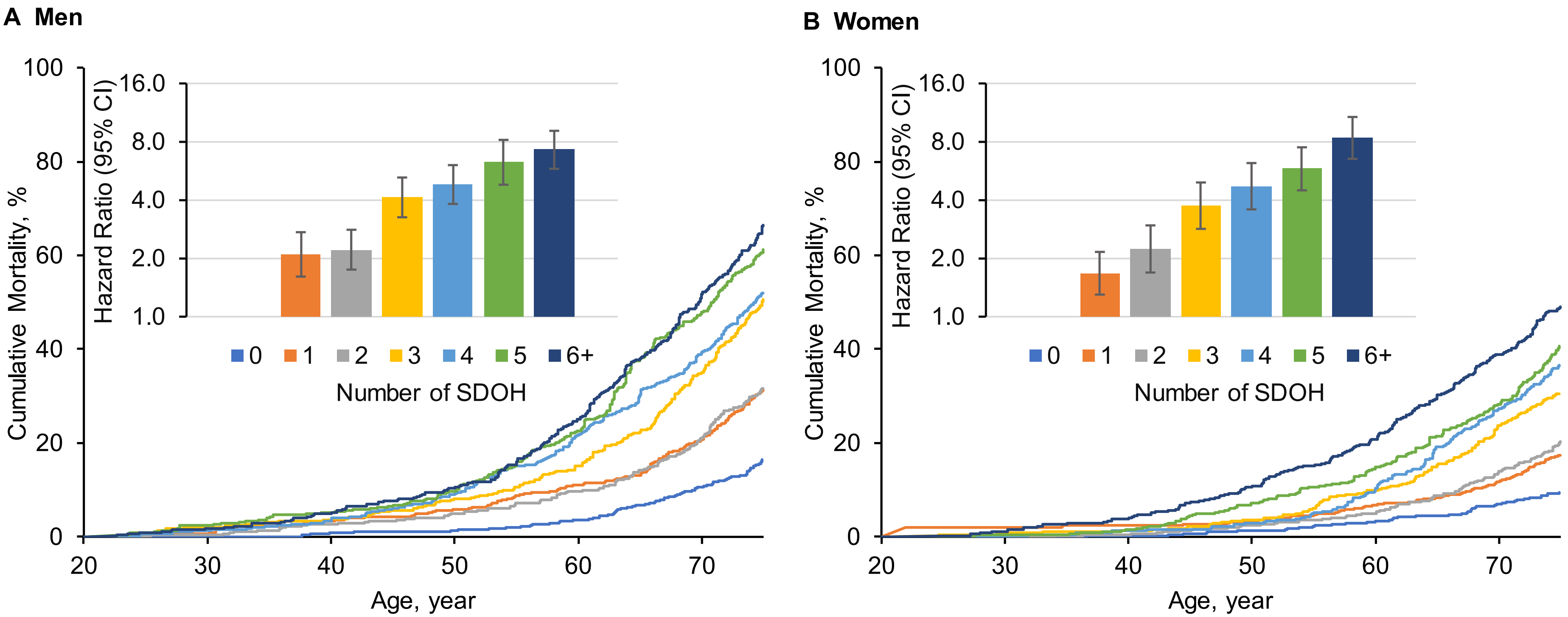

Kaplan-Meier curves show cumulative mortality rates by age and number of unfavorable social determinants of health (SDOH). Bar charts show age-, and race/ethnicity-adjusted hazard ratios and 95% confidence intervals of premature all-cause mortality associated with number of unfavorable SDOH. (A) Among men (n=23,176), compared with those with 0 unfavorable SDOH, hazard ratios (95% CIs) for men with 1, 2, 3, 4, 5, or ≥6 unfavorable SDOH were 2·10 (1·61-2·73), 2·22 (1·75-2·81), 4·13 (3·26-5·23), 4·81 (3·82-6·06), 6·27 (4·80-8·18), and 7·27 (5·80-9·11), respectively (p<0·0001 for linear trend). (B) Among women (n=24,994), compared with those with 0 unfavorable SDOH, hazard ratios (95% CIs) for women with 1, 2, 3, 4, 5, or ≥6 unfavorable SDOH were 1·67 (1·30-2·16), 2·23 (1·69-2·96), 3·75 (2·84-4·93), 4·72 (3·58-6·21), 5·80 (4·49-7·49), and 8·37 (6·53-10·74), respectively (p<0·0001 for linear trend).

Table S1: US Centers for Disease Control Healthy People 2030 Social Determinants of Health in NHANES 1999-2018.

Domain	Subdomain	Questions and Definitions
**Economic Stability**	Employment	Participants were asked what type of work they had done in the last week and, if not working, the main reason why. All participants not working were classified as unemployed, except those who responded they were a student or retired were grouped with those reporting employment.
	Poverty/income ratio	Participants were asked their family income and size, and the poverty/income ratio (PIR) is the ratio of family income to poverty. The Department of Health and Human Services poverty guidelines were used as the poverty measure to calculate this ratio. These guidelines are issued each year, in the Federal Register, for determining financial eligibility for certain federal programs. The guidelines vary by family size and geographic location.
	Food security	Participants responded to the U.S. Food Security Survey Module questions (Bickel G, et al, 2000) whether: 1) they were worried if food would run out before there was money to buy more; 2) the food they bought didn't last and they didn't have money to get more; 3) they couldn't afford to eat balanced meals; 4) they had cut the size of meals or skipped meals because there wasn't enough money for food; 5) if yes to #4, how often meals were cut or skipped; 6) they ate less than they felt they should because there was not enough money to buy food; 7) they were hungry but didn't eat because they couldn't afford food; 8) they lost weight because they didn't have enough money for food; 9) they did not eat for a whole day because there was not enough money for food; 10) if yes to #9, how often they did not eat for the whole day. Levels of food security were classified as follows: full food security, no affirmative responses; marginal food security, 1-2 affirmative responses; low food security, 3-5 affirmative responses; and very low food security, 6-10 affirmative responses.
**Education Access and Quality**	Education level	Participants were asked for the highest grade or level of school they completed. The response categories are: less than 9th grade education, 9-11th grade education (includes 12th grade and no diploma), High school graduate/GED, some college or associates (AA) degree, and college graduate or higher.
**Healthcare Access and Quality**	Access to healthcare	Participants were asked if there is a place they usually go when sick or needing advice about health. If they answered "yes" or "there is more than one place" to this question, they were classified as having a routine place for healthcare. If yes, but the facility is a hospital emergency room, they were classified as not having a routine place for healthcare.
	Health insurance	Participants were asked whether they are covered by health insurance or some other kind of health care plan. They are subsequently asked if covered by private insurance or several types of government insurance (Medicare, Medi-Gap, Medicaid, SCHIP, military health care, Indian Health Service, state-sponsored health plan, or other government insurance).
**Neighborhood and Built Environment**	Housing instability	Participants were asked if the home they are living in is owned, being bought, rented, or occupied by some other arrangement. A person was considered to own the home even if they are still paying on a mortgage.
**Social and Community Context**	Marital status	Participants were asked whether they were married, widowed, divorced, separated, never married, or living with a partner. Those reporting marriage or living with a partner were grouped together.

NHANES denotes National Health and Nutrition Examination Survey

Table S2: Candidate Social Determinants of Health and Associations with Premature All-cause Mortality in US Adults Aged 20 to 74 Years, NHANES 1999-2018.

Domain	Subdomain/Question	Categories and Coding	Hazard Ratio (95% CI) *
Economic Stability	Employment	0: Employed, student, retired<br>1: Not employed	[Reference]<br>3·06 (2·79-3·36)
	Poverty/income ratio	1: 500% ≤ PIR<br>2: 300% ≤ PIR < 500%<br>3: 100% < PIR < 300%<br>4: PIR ≤ 100%	[Reference]<br>1·60 (1·36-1·88)<br>2·83 (2·46-3·26)<br>4·68 (3·97-5·52)
	Poverty/income ratio (dichotomized at median)	0: PIR ≥ 300%<br>1: PIR < 300%	[Reference]<br>2·55 (2·27-2·85)
	Food security (10-item)	1: Full food security (0 affirmative)<br>2: Marginal (1-2 affirmative)<br>3: Low (3-5 affirmative)<br>4: Very low (6-10 affirmative)	[Reference]<br>1·96 (1·71-2·24)<br>2·03 (1·74-2·37)<br>2·81 (2·43-3·25)
	Food security (10-item, dichotomized)	0: Full food security<br>1: Marginal, low, or very low	[Reference]<br>2·20 (2·00-2·43)
Education Access and Quality	Education level	1: College<br>2: Some college<br>3: Highschool<br>4: Less than high school	[Reference]<br>1·95 (1·66-2·30)<br>2·24 (1·89-2·65)<br>3·55 (3·00-4·20)
	High school graduate	0: High school or more<br>1: Less than high school	[Reference]<br>2·06 (1·82-2·33)
	College graduate	0: College graduate<br>1: Less than college graduate	[Reference]<br>2·37 (2·05-2·75)
Healthcare Access and Quality	Access to healthcare	0: Routine place to go for healthcare<br>1: No routine place, or ER/hospital/other	[Reference]<br>1·29 (1·13-1·48)
	Health insurance	1: Private insurance<br>2: Government insurance<br>3: No insurance	[Reference]<br>2·84 (2·52-3·19)<br>1·90 (1·66-2·17)
	Health insurance (dichotomized)	0: Private insurance<br>1: Government or no insurance	[Reference]<br>2·40 (2·17-2·67)
Neighborhood and Built Environment	Housing instability	0: Own home<br>1: Rent or other arrangement	[Reference]<br>1·72 (1·55-1·91)
Social and Community Context	Marital status	0: Married or living with a partner<br>1: Not married nor living with a partner	[Reference]<br>1·92 (1·74-2·12)

* Models were stratified by birth cohort and adjusted for gender and race/ethnicity.
NHANES denotes National Health and Nutrition Examination Survey

Table S3. Pairwise Correlations between Social Determinants of Health in US Adults Aged 20 to 74 Years, NHANES 1999-2018.*

Social Determinants of Health	1	2	3	4	5	6	7	8
1. Employment status	1·0000							
2. Family income-to-poverty ratio	0·2244	1·0000						
3. Food security	0·1896	0·3952	1·0000					
4. Education level	0·1644	0·2831	0·2079	1·0000				
5. Regular health care access	0·0118	0·1838	0·1547	0·1110	1·0000			
6. Type of health insurance	0·2874	0·4338	0·3181	0·2681	0·2445	1·0000		
7. Home ownership	0·0958	0·3431	0·2922	0·1383	0·2027	0·2669	1·0000	
8. Marital status	0·0498	0·1900	0·1192	0·0255	0·1196	0·1785	0·2417	1·0000

* Correlations were estimated using Cramér's V for dichotomous variables. All pairwise correlations between social determinants of health are statistically significant (p<0·001).

Table S4. Associations of Social Determinants of Health with Premature All-cause Mortality in US Adults Aged 20 to 74 Years by Race/ethnicity, NHANES 1999-2018.

Social Determinants of Health	Black, Hazard Ratio (95% CI)		Hispanic, Hazard Ratio (95% CI)		White, Hazard Ratio (95% CI)		Other, Hazard Ratio (95% CI)	
	Model 1 *	Model 2 †	Model 1 *	Model 2 †	Model 1 *	Model 2 †	Model 1 *	Model 2 †
Employment status								
Employed, student, or retired	Reference	Reference	Reference	Reference	Reference	Reference	Reference	Reference
Unemployed	2·93 (2·54-3·38)	2·21 (1·87-2·61)	1·98 (1·61-2·43)	1·87 (1·53-2·29)	3·38 (2·97-3·85)	2·16 (1·86-2·50)	2·14 (1·45-3·17)	1·66 (1·10-2·50)
Family income-to-poverty ratio								
≥300%	Reference	Reference	Reference	Reference	Reference	Reference	Reference	Reference
<300%	2·35 (1·99-2·78)	1·54 (1·27-1·86)	1·43 (1·09-1·86)	1·27 (0·90-1·79)	2·57 (2·27-2·91)	1·41 (1·21-1·65)	2·40 (1·68-3·44)	1·66 (1·04-2·63)
Food security								
Full security	Reference	Reference	Reference	Reference	Reference	Reference	Reference	Reference
Marginal, low, or very low security	1·50 (1·30-1·72)	0·97 (0·84-1·12)	1·27 (1·05-1·54)	1·08 (0·87-1·34)	2·70 (2·38-3·07)	1·31 (1·12-1·52)	2·41 (1·61-3·61)	1·50 (1·01-2·24)
Education level								
High school graduate or higher	Reference	Reference	Reference	Reference	Reference	Reference	Reference	Reference
Less than high school	1·72 (1·48-1·98)	1·22 (1·04-1·42)	1·09 (0·89-1·33)	0·96 (0·77-1·19)	2·48 (2·08-2·95)	1·50 (1·25-1·80)	1·59 (0·95-2·66)	1·12 (0·66-1·89)
Regular health care access								
≥One regular health care facility	Reference	Reference	Reference	Reference	Reference	Reference	Reference	Reference
None or emergency room	1·21 (1·02-1·42)	0·96 (0·82-1·13)	0·84 (0·64-1·09)	0·79 (0·61-1·02)	1·40 (1·18-1·67)	1·01 (0·85-1·21)	1·62 (0·99-2·67)	1·35 (0·81-2·27)
Type of health insurance								
Private	Reference	Reference	Reference	Reference	Reference	Reference	Reference	Reference
Government or none	2·15 (1·85-2·49)	1·30 (1·09-1·55)	1·24 (0·98-1·58)	0·95 (0·72-1·26)	2·70 (2·37-3·07)	1·47 (1·25-1·74)	1·63 (1·17-2·29)	0·88 (0·60-1·30)
Home ownership								
Own home	Reference	Reference	Reference	Reference	Reference	Reference	Reference	Reference
Rent home or other arrangement	1·45 (1·22-1·72)	0·99 (0·83-1·18)	1·20 (0·97-1·49)	1·02 (0·80-1·29)	1·90 (1·68-2·16)	1·05 (0·92-1·20)	1·80 (1·18-2·76)	1·27 (0·86-1·88)
Marital status								
Married or living with a partner	Reference	Reference	Reference	Reference	Reference	Reference	Reference	Reference
Not married nor living with a partner	1·54 (1·31-1·81)	1·18 (0·99-1·40)	1·78 (1·28-2·46)	1·75 (1·23-2·49)	1·98 (1·76-2·23)	1·41 (1·25-1·60)	2·17 (1·45-3·25)	1·78 (1·16-2·73)

* Model 1 was stratified by birth cohort and adjusted for gender.

† Model 2 was stratified by birth cohort, and adjusted for gender and all social determinants of health in this table.

Table S5. Associations of Social Determinants of Health with Premature All-cause Mortality in US Adults Aged 20 to 74 Years by Cause of Death, NHANES 1999-2018.

Social Determinants of Health	Cardiovascular Disease Death, Hazard Ratio (95% CI)		Cancer Death, Hazard Ratio (95% CI)		Non-Cardiovascular and Non-Cancer Death, Hazard Ratio (95% CI)	
	**Model 1 ***	**Model 2 †**	**Model 1 ***	**Model 2 †**	**Model 1 ***	**Model 2 †**
Employment status						
Employed, student, or retired	Reference	Reference	Reference	Reference	Reference	Reference
Unemployed	3·35 (2·74-4·10)	2·14 (1·72-2·66)	2·16 (1·78-2·61)	1·62 (1·31-1·99)	3·60 (3·21-4·04)	2·44 (2·16-2·76)
Family income-to-poverty ratio						
≥300%	Reference	Reference	Reference	Reference	Reference	Reference
<300%	2·83 (2·27-3·55)	1·61 (1·24-2·09)	1·82 (1·51-2·20)	1·23 (0·99-1·54)	2·82 (2·45-3·25)	1·62 (1·38-1·91)
Food security						
Full security	Reference	Reference	Reference	Reference	Reference	Reference
Marginal, low, or very low security	2·40 (1·97-2·93)	1·20 (0·97-1·50)	1·61 (1·33-1·96)	1·02 (0·82-1·27)	2·45 (2·14-2·81)	1·29 (1·12-1·49)
Education level						
High school graduate or higher	Reference	Reference	Reference	Reference	Reference	Reference
Less than high school	2·40 (1·99-2·88)	1·50 (1·24-1·82)	1·99 (1·60-2·49)	1·51 (1·20-1·91)	1·95 (1·62-2·34)	1·18 (0·99-1·41)
Regular health care access						
≥One regular health care facility	Reference	Reference	Reference	Reference	Reference	Reference
None or emergency room	1·17 (0·89-1·53)	0·85 (0·64-1·12)	1·33 (1·04-1·70)	1·07 (0·82-1·38)	1·33 (1·11-1·59)	0·99 (0·84-1·17)
Type of health insurance						
Private	Reference	Reference	Reference	Reference	Reference	Reference
Government or none	2·53 (1·97-3·24)	1·32 (0·98-1·77)	1·95 (1·64-2·33)	1·35 (1·09-1·67)	2·63 (2·29-3·02)	1·37 (1·17-1·61)
Home ownership						
Own home	Reference	Reference	Reference	Reference	Reference	Reference
Rent home or other arrangement	2·08 (1·76-2·46)	1·20 (1·01-1·42)	1·35 (1·11-1·64)	0·91 (0·74-1·13)	1·80 (1·56-2·08)	1·06 (0·92-1·22)
Marital status						
Married or living with a partner	Reference	Reference	Reference	Reference	Reference	Reference
Not married nor living with a partner	2·12 (1·71-2·64)	1·51 (1·18-1·94)	1·65 (1·39-1·95)	1·38 (1·17-1·63)	1·97 (1·70-2·29)	1·46 (1·25-1·69)

* Model 1 was stratified by birth cohort and adjusted for gender.

† Model 2 was stratified by birth cohort, and adjusted for gender and all social determinants of health in this table.

Table S6. Mediation of the Hispanic-White Difference in Premature Death by Social Determinants of Health in US Adults Aged 20 to 74 Years, National Health and Nutrition Examination Surveys 1999-2018.

Characteristics	Hazard Ratio (95% CI)	Relative Contribution, % (95% CI)*
Stratified by birth cohort and adjusted for gender		
Hispanic vs. White race	0·84 (0·72-0·98)	--
Stratified by birth cohort and adjusted for gender and other SDOH		
Hispanic vs. White race	0·50 (0·42-0·60)	--
Unemployed	2·14 (1·88-2·44)	-22·8 (-40·8, -4·9)
Family income-to-poverty ratio <300%	1·43 (1·24-1·65)	-51·2 (-93·0, -9·3)
Marginal, low, or very low food security	1·25 (1·10-1·43)	-25·9 (-46·3, -5·5)
Less than high school education	1·39 (1·18-1·64)	-42·8 (-74·4, -11·2)
No regular health care access	0·96 (0·82-1·13)	3·0 (-9·4, 15·3)
No private health insurance	1·40 (1·20-1·64)	-43·1 (-74·8, -11·4)
Not own home	1·04 (0·92-1·17)	-3·8 (-17·1, 9·6)
Not married nor living with a partner	1·48 (1·32-1·66)	-5·4 (-10·9, 0·1)

*The relative contribution is the percentage of the ethnicity difference in all-cause mortality explained by each factor in the multivariable mediation model. SDOH denotes social determinants of health.

Table S7. Characteristics of Study Participants Aged 20 Years or Older by Race/ethnicity, NHANES 1999-2018. *

Characteristics	All Participants (N = 54927)	Self-reported Race/Ethnicity †				P-values for Group Difference
		Black (N = 11479)	Hispanic (N = 14083)	White (N = 24254)	Other (N = 5111)	
Age, mean (95% CI), y	47·0 (46·7-47·4)	44·5 (44·1-45·0)	41·0 (40·5-41·6)	49·0 (48·6-49·4)	44·3 (43·6-45·0)	<0·001
Gender, % (95% CI)						
Women	52·0 (51·6-52·4)	55·4 (54·6-56·2)	50·0 (49·2-50·9)	51·7 (51·2-52·3)	52·6 (51·1-54·2)	<0·001
Men	48·0 (47·6-48·4)	44·6 (43·8-45·4)	50·0 (49·1-50·8)	48·3 (47·7-48·8)	47·4 (45·8-48·9)	
Employment status, % (95% CI)						
Employed, student, or retired	80·6 (80·0-81·3)	74·4 (73·1-75·7)	75·0 (74·0-76·1)	83·1 (82·2-83·9)	77·9 (76·4-79·5)	<0·001
Unemployed	19·4 (18·7-20·0)	25·6 (24·3-26·9)	25·0 (23·9-26·0)	16·9 (16·1-17·8)	22·1 (20·5-23·6)	
Family income-to-poverty ratio, % (95% CI)						
≥300%	49·0 (47·6-50·5)	32·3 (30·4-34·3)	24·4 (22·8-26·0)	56·9 (55·0-58·7)	48·0 (44·9-51·0)	<0·001
<300%	51·0 (49·5-52·4)	67·7 (65·7-69·6)	75·6 (74·0-77·2)	43·1 (41·3-45·0)	52·0 (49·0-55·1)	
Food security, % (95% CI) ‡						
Full security	78·9 (78·0-79·8)	65·7 (64·0-67·4)	57·8 (55·9-59·7)	85·4 (84·5-86·3)	78·6 (76·4-80·8)	<0·001
Marginal, low, or very low security	21·1 (20·2-22·0)	34·3 (32·6-36·0)	42·2 (40·3-44·1)	14·6 (13·7-15·5)	21·4 (19·2-23·6)	
Education level, % (95% CI)						
High school graduate or higher	82·4 (81·5-83·2)	75·8 (74·4-77·2)	57·6 (56·1-59·1)	88·3 (87·2-89·4)	84·5 (82·9-86·1)	<0·001
Less than high school	17·6 (16·8-18·5)	24·2 (22·8-25·6)	42·4 (40·9-43·9)	11·7 (10·6-12·8)	15·5 (13·9-17·1)	
Regular health care access, % (95% CI)						
≥One regular health care facility	80·0 (79·2-80·7)	76·3 (75·1-77·6)	65·6 (64·0-67·2)	84·0 (83·2-84·8)	74·8 (72·9-76·8)	<0·001
None or regular facility is emergency room	20·0 (19·3-20·8)	23·7 (22·4-24·9)	34·4 (32·8-36·0)	16·0 (15·2-16·8)	25·2 (23·2-27·1)	
Type of health insurance, % (95% CI)						
Private	63·3 (62·2-64·4)	49·8 (48·1-51·4)	40·6 (39·1-42·1)	70·6 (69·4-71·9)	59·0 (56·5-61·5)	<0·001
Government or none	36·7 (35·6-37·8)	50·2 (48·6-51·9)	59·4 (57·9-60·9)	29·4 (28·1-30·6)	41·0 (38·5-43·5)	

Characteristics	All Participants (N = 54927)	Self-reported Race/Ethnicity †				P-values for Group Difference
		Black (N = 11479)	Hispanic (N = 14083)	White (N = 24254)	Other (N = 5111)	
Home ownership, % (95% CI)						
Own home	67·7 (66·4-69·0)	48·2 (46·2-50·2)	50·4 (47·9-53·0)	75·3 (74·1-76·5)	60·2 (57·0-63·5)	<0·001
Rent home or other arrangement	32·3 (31·0-33·6)	51·8 (49·8-53·8)	49·6 (47·0-52·1)	24·7 (23·5-25·9)	39·8 (36·5-43·0)	
Marital status, % (95% CI)						
Married or living with a partner	63·1 (62·2-64·1)	43·4 (41·9-44·8)	63·7 (62·3-65·1)	66·1 (65·1-67·1)	65·6 (63·6-67·6)	<0·001
Not married nor living with a partner	36·9 (35·9-37·8)	56·6 (55·2-58·1)	36·3 (34·9-37·7)	33·9 (32·9-34·9)	34·4 (32·4-36·4)	
Aggregate SDOH score, % (95% CI)						
0	20·9 (20·0-21·9)	8·8 (7·9-9·8)	7·0 (6·3-7·7)	26·2 (25·0-27·4)	16·4 (14·5-18·3)	<0·001
1	20·3 (19·6-21·1)	12·7 (11·8-13·5)	10·0 (9·2-10·8)	23·9 (23·0-24·8)	18·5 (16·6-20·5)	
2	16·4 (15·9-17·0)	14·3 (13·6-15·1)	12·4 (11·6-13·2)	17·4 (16·7-18·1)	18·3 (16·6-19·9)	
3	13·8 (13·3-14·2)	15·6 (14·8-16·4)	15·2 (14·4-16·1)	12·9 (12·3-13·5)	16·0 (14·6-17·4)	
4	12·1 (11·6-12·5)	17·8 (16·9-18·6)	17·9 (17·1-18·8)	9·7 (9·2-10·3)	14·2 (12·6-15·7)	
5	9·1 (8·6-9·5)	15·4 (14·5-16·4)	18·4 (17·4-19·4)	6·0 (5·6-6·5)	9·9 (8·8-11·0)	
6 or more	7·4 (7·0-7·8)	15·3 (14·2-16·4)	19·1 (17·8-20·3)	3·8 (3·4-4·2)	6·8 (5·7-7·9)	

* The sample size for each race/ethnicity group is unweighted, but all other numbers in the table are weighted means or percentages (with 95% confidence intervals). NHANES denotes the National Health and Nutrition Examination Survey.

† Other includes non-Hispanic Asian, American Indian or Alaska Native, and Native Hawaiian or Pacific Islander.

‡ Full food security indicates 0 affirmative responses to the individual Food Security Survey Module questions, and marginal or lower food security indicates one or more affirmative responses.

Table S8. Associations of Social Determinants of Health with All-cause Mortality in US Adults Aged 20 Years or Older, NHANES 1999-2018.

Social Determinants of Health	Deaths / Total No·	Model 1 HR (95% CI) *	Model 2 HR (95% CI) †
Employment status			
Employed, student, or retired	6961 / 42166	Reference	Reference
Unemployed	2200 / 12706	2·27 (2·11-2·44)	1·86 (1·73-2·00)
Family income-to-poverty ratio			
≥300%	2166 / 18371	Reference	Reference
<300%	6013 / 31302	1·77 (1·66-1·88)	1·35 (1·26-1·44)
Food security			
Full security	7164 / 38621	Reference	Reference
Marginal, low, or very low security	1758 / 14804	1·78 (1·66-1·92)	1·23 (1·15-1·31)
Education level			
High school graduate or higher	5413 / 39700	Reference	Reference
Less than high school	3703 / 15109	1·50 (1·40-1·61)	1·17 (1·09-1·26)
Regular health care access			
≥One regular health care facility	8089 / 43115	Reference	Reference
None or emergency room	1085 / 11807	1·26 (1·14-1·39)	1·05 (0·95-1·15)
Type of health insurance			
Private	4232 / 28908	Reference	Reference
Government or none	4810 / 25373	1·57 (1·48-1·67)	1·18 (1·11-1·25)
Home ownership			
Own home	6398 / 33532	Reference	Reference
Rent home or other arrangement	2645 / 20355	1·50 (1·42-1·59)	1·12 (1·05-1·19)
Marital status			
Married or living with a partner	4511 / 32353	Reference	Reference
Not married nor living with a partner	4471 / 21985	1·56 (1·48-1·64)	1·33 (1·26-1·40)

* Model 1 was stratified by birth cohort and adjusted for gender and race/ethnicity.

† Model 2 was stratified by birth cohort, and adjusted for gender, race/ethnicity, and all social determinants of health in this table.

Table S9. Associations of Social Determinants of Health with Premature All-cause Mortality in US Adults by Varying Age Cut-off Points, NHANES 1999-2018.

Social Determinants of Health	Death Before Age 65, Hazard Ratio (95% CI)		Death Before Age 70, Hazard Ratio (95% CI)		Death Before Age 75, Hazard Ratio (95% CI)		Death Before Age 80, Hazard Ratio (95% CI)	
	Model 1 *	Model 2 †	Model 1 *	Model 2 †	Model 1 *	Model 2 †	Model 1 *	Model 2 †
Employment status								
Employed, student, or retired	Reference	Reference	Reference	Reference	Reference	Reference	Reference	Reference
Unemployed	3·31 (2·95-3·72)	2·12 (1·89-2·39)	3·13 (2·82-3·47)	2·08 (1·87-2·31)	3·06 (2·79-3·36)	2·11 (1·91-2·33)	2·86 (2·64-3·10)	2·08 (1·91-2·27)
Family income-to-poverty ratio								
≥300%	Reference	Reference	Reference	Reference	Reference	Reference	Reference	Reference
<300%	2·62 (2·29-3·00)	1·39 (1·19-1·62)	2·60 (2·29-2·95)	1·49 (1·29-1·73)	2·45 (2·21-2·73)	1·48 (1·31-1·67)	2·31 (2·11-2·53)	1·51 (1·37-1·68)
Food security								
Full security	Reference	Reference	Reference	Reference	Reference	Reference	Reference	Reference
Marginal, low, or very low security	2·38 (2·10-2·70)	1·31 (1·14-1·50)	2·28 (2·04-2·55)	1·25 (1·10-1·41)	2·18 (1·98-2·40)	1·20 (1·09-1·33)	2·11 (1·94-2·29)	1·22 (1·12-1·33)
Education level								
High school graduate or higher	Reference	Reference	Reference	Reference	Reference	Reference	Reference	Reference
Less than high school	2·02 (1·72-2·38)	1·28 (1·09-1·49)	2·05 (1·79-2·35)	1·31 (1·15-1·49)	2·05 (1·81-2·32)	1·33 (1·18-1·51)	1·89 (1·70-2·11)	1·28 (1·15-1·43)
Regular health care access								
≥One regular health care facility	Reference	Reference	Reference	Reference	Reference	Reference	Reference	Reference
None or emergency room	1·34 (1·14-1·58)	0·98 (0·84-1·14)	1·32 (1·14-1·53)	0·98 (0·85-1·14)	1·29 (1·13-1·48)	0·98 (0·86-1·11)	1·29 (1·14-1·46)	1·01 (0·89-1·13)
Type of health insurance								
Private	Reference	Reference	Reference	Reference	Reference	Reference	Reference	Reference
Government or none	3·00 (2·58-3·48)	1·61 (1·35-1·92)	2·64 (2·32-3·00)	1·43 (1·22-1·66)	2·38 (2·15-2·64)	1·35 (1·20-1·53)	2·06 (1·88-2·25)	1·24 (1·12-1·37)
Home ownership								
Own home	Reference	Reference	Reference	Reference	Reference	Reference	Reference	Reference
Rent home or other arrangement	1·64 (1·45-1·86)	0·98 (0·87-1·10)	1·69 (1·51-1·89)	1·02 (0·91-1·13)	1·71 (1·55-1·90)	1·05 (0·95-1·15)	1·68 (1·54-1·83)	1·06 (0·97-1·16)
Marital status								
Married or living with a partner	Reference	Reference	Reference	Reference	Reference	Reference	Reference	Reference
Not married nor living with a partner	1·96 (1·71-2·24)	1·47 (1·28-1·70)	1·93 (1·73-2·16)	1·45 (1·29-1·63)	1·90 (1·73-2·08)	1·44 (1·32-1·58)	1·80 (1·66-1·95)	1·40 (1·30-1·52)

* Model 1 was stratified by birth cohort and adjusted for gender.
† Model 2 was stratified by birth cohort, and adjusted for gender and all social determinants of health in this table.

Table S10: Characteristics of Study Participants Aged 20 to 74 Years by 10-year Survey Cycle Groups, NHANES 1999-2018.

Characteristics	Overall (N = 48170)	10-year NHANES Survey Cycles	
		1999-2008 (N = 22587)	2009-2018 (N = 25583)
Age, mean (95% CI), y	44·3 (44·0-44·6)	43·6 (43·2-44·0)	45·0 (44·6-45·5)
Gender, % (95% CI)			
Women	51·3 (50·9-51·8)	51·3 (50·8-51·9)	51·3 (50·7-52·0)
Men	48·7 (48·2-49·1)	48·7 (48·1-49·2)	48·7 (48·0-49·3)
Race/ethnicity, % (95% CI)			
Non-Hispanic Black	11·6 (10·4-12·7)	11·4 (9·8-13·0)	11·7 (10·1-13·2)
Hispanic	14·5 (13·0-16·0)	13·4 (11·4-15·4)	15·5 (13·3-17·8)
Non-Hispanic White	66·7 (64·6-68·7)	69·7 (67·0-72·4)	64·0 (60·9-67·0)
Other	7·2 (6·6-7·9)	5·5 (4·7-6·2)	8·8 (7·8-9·8)
Employment status, % (95% CI)			
Employed, student, or retired	79·8 (79·1-80·5)	80·6 (79·7-81·6)	79·1 (78·1-80·1)
Unemployed	20·2 (19·5-20·9)	19·4 (18·4-20·3)	20·9 (19·9-21·9)
Family income-to-poverty ratio, % (95% CI)			
≥300%	50·2 (48·7-51·7)	51·4 (49·3-53·5)	49·2 (47·0-51·3)
<300%	49·8 (48·3-51·3)	48·6 (46·5-50·7)	50·8 (48·7-53·0)
Food security, % (95% CI) *			
Full security	78·0 (77·1-78·9)	83·3 (82·4-84·3)	73·2 (71·7-74·7)
Marginal, low, or very low security	22·0 (21·1-22·9)	16·7 (15·7-17·6)	26·8 (25·3-28·3)
Education level, % (95% CI)			
High school graduate or higher	83·3 (82·5-84·2)	81·1 (80·0-82·2)	85·3 (84·1-86·6)
Less than high school	16·7 (15·8-17·5)	18·9 (17·8-20·0)	14·7 (13·4-15·9)
Regular health care access, % (95% CI)			
≥One regular health care facility	78·8 (78·0-79·6)	80·1 (79·1-81·2)	77·6 (76·5-78·7)

Characteristics	Overall (N = 48170)	10-year NHANES Survey Cycles	
		1999-2008 (N = 22587)	2009-2018 (N = 25583)
None or emergency room	21·2 (20·4-22·0)	19·9 (18·8-20·9)	22·4 (21·3-23·5)
Type of health insurance, % (95% CI)			
Private	63·9 (62·8-65·0)	66·8 (65·3-68·3)	61·3 (59·6-63·0)
Government or none	36·1 (35·0-37·2)	33·2 (31·7-34·7)	38·7 (37·0-40·4)
Home ownership, % (95% CI)			
Own home	66·7 (65·4-68·1)	69·1 (67·2-71·0)	64·6 (62·8-66·5)
Rent home or other arrangement	33·3 (31·9-34·6)	30·9 (29·0-32·8)	35·4 (33·5-37·2)
Marital status, % (95% CI)			
Married or living with a partner	64·4 (63·4-65·3)	65·3 (64·0-66·7)	63·5 (62·2-64·8)
Not married nor living with a partner	35·6 (34·7-36·6)	34·7 (33·3-36·0)	36·5 (35·2-37·8)
Aggregate SDOH score, % (95% CI)			
0	21·8 (20·8-22·9)	23·4 (22·0-24·9)	20·4 (18·9-21·8)
1	20·1 (19·4-20·8)	20·9 (19·9-21·9)	19·4 (18·4-20·5)
2	15·8 (15·2-16·3)	16·1 (15·4-16·9)	15·4 (14·6-16·3)
3	13·3 (12·8-13·8)	13·3 (12·6-14·0)	13·3 (12·6-13·9)
4	12·0 (11·5-12·5)	11·2 (10·5-11·8)	12·7 (12·0-13·4)
5	9·2 (8·8-9·7)	8·1 (7·5-8·7)	10·3 (9·5-11·0)
6 or more	7·8 (7·3-8·2)	6·9 (6·4-7·4)	8·5 (7·8-9·2)

* Full food security indicates 0 affirmative responses to the individual Food Security Survey Module questions, and marginal or lower food security indicates one or more affirmative responses.

Table S11: Associations of Social Determinants of Health with Premature All-cause Mortality in US Adults Aged 20 to 74 Years by 10-year Survey Cycle Groups, NHANES 1999-2018.

Social Determinants of Health	1999-2008, Hazard Ratio (95% CI)		2009-2018, Hazard Ratio (95% CI)	
	Model 1 *	Model 2 †	Model 1 *	Model 2 †
Employment status				
Employed, student, or retired	Reference	Reference	Reference	Reference
Unemployed	2·88 (2·58-3·23)	1·99 (1·76-2·26)	3·47 (2·95-4·07)	2·32 (1·95-2·75)
Family income-to-poverty ratio				
≥300%	Reference	Reference	Reference	Reference
<300%	2·38 (2·10-2·70)	1·48 (1·29-1·70)	2·75 (2·25-3·36)	1·49 (1·15-1·93)
Food security				
Full security	Reference	Reference	Reference	Reference
Marginal, low, or very low security	1·98 (1·76-2·23)	1·09 (0·98-1·21)	2·68 (2·27-3·18)	1·48 (1·19-1·84)
Education level				
High school graduate or higher	Reference	Reference	Reference	Reference
Less than high school	2·13 (1·84-2·48)	1·43 (1·23-1·66)	1·81 (1·45-2·26)	1·08 (0·87-1·34)
Regular health care access				
≥One regular health care facility	Reference	Reference	Reference	Reference
None or emergency room	1·30 (1·11-1·53)	1·01 (0·86-1·17)	1·29 (1·00-1·65)	0·90 (0·70-1·14)
Type of health insurance				
Private	Reference	Reference	Reference	Reference
Government or none	2·31 (2·04-2·63)	1·33 (1·14-1·54)	2·74 (2·32-3·24)	1·51 (1·24-1·84)
Home ownership				
Own home	Reference	Reference	Reference	Reference
Rent home or other arrangement	1·70 (1·50-1·93)	1·08 (0·96-1·21)	1·75 (1·49-2·07)	0·97 (0·82-1·14)
Marital status				
Married or living with a partner	Reference	Reference	Reference	Reference
Not married nor living with a partner	1·84 (1·64-2·07)	1·42 (1·27-1·59)	2·07 (1·78-2·40)	1·49 (1·28-1·73)

* Model 1 was stratified by birth cohort and adjusted for gender and race/ethnicity.

† Model 2 was stratified by birth cohort, and adjusted for gender, race/ethnicity, and all social determinants of health in this table.
